# Supplementary material for: Estimating psychophysiological loads by repeated temperature steps on humans using a state–space model
Source: PLoS One. 2025 Nov 3;20(11):e0335545. doi: 10.1371/journal.pone.0335545 (PMC12582479; doi:10.1371/journal.pone.0335545)
Supplement: Attachment — Submitted filename: Response To Reviewers_PONE-D-25-06325.docx [file pone.0335545.s013.docx]

Responses to the Editor’s and Reviewers’ Comments

Dear Dr. Neelu Jain Gupta, Academic Editor of PLOS ONE,

The authors would like to thank the Editor and the Reviewers for generously providing the comments on our manuscript (PONE-D-25-06325: Estimating Psychophysiological Loads by Repeated Temperature Steps on Humans using a State–Space Model) by Miho Iwasaki, Yusuke Morito, Kyosuke Watanabe, Kiyoshi Kuroi, Shota Hori, Yoko Sakata, Kei Mizuno, Kazunobu Okazaki and Yasuyoshi Watanabe. Here, we would like to send the revised version and point-by-point responses to each of the comments made by the Editor and Reviewers as detailed on the following pages.

Thank you so much again for the Editor’s and Reviewers’ generous comments on our manuscript. We do hope that the revised manuscript and our point-by-point responses to the Reviewers could be generously understood by the Editor and Reviewers, and now the revised version will be acceptable for publication in PLoS One.

Cordially yours,

**Y. Watanabe**

**Yasuyoshi Watanabe, MD, PhD**

**Honorary Scientist, RIKEN**

**Phone: +81-78-304-7101**

**Responses to Additional Editor Comments:**

[Part 1]

Linking daily temperature variations between indoors and outdoors to health is pertinent worldwide. The study requires significant adjustments because its current methodology is very constrained 1) sample size specially when it addresses psychophysiological burdens.

**[Response]**

**We sincerely thank the Editor for the thoughtful and constructive comments regarding our manuscript. As this study was conducted in a tightly controlled laboratory environment, our primary aim was to explore psychophysiological responses to short-term temperature changes under experimental conditions. We fully agree that further research with a larger sample size and in real-world environments will be essential to improve generalizability and assess broader health implications. To address this, we have added a Limitation section in the revised manuscript, where we discuss this issue in detail (see lines 418–430).**

[Part 2]

The planned study is fine, however the physical exhaustion scale is too broad (0–100), making assessments more arbitrary.

**[Response]**

**We thank the editor’s comment on the use of a 0–100 visual analogue scale (VAS) for assessing physical fatigue. The VAS is a widely used and validated tool for capturing subjective fatigue, as shown by Lee et al. (1991). Its continuous format allows for detecting subtle changes that may be missed by categorical scales, making it suitable for psychophysiological studies like ours. To minimize variability, we gave participants clear instructions with anchor definitions (0 = no fatigue, 100 = complete exhaustion) and ensured consistency across sessions. We have also revised the Methods section to better explain our use of the VAS (see lines 112–118).**

[Part 3]

One reviewer has rightly suggested that tests such as as skin temperature, ECG, HR, and HRV, factors like activity and sleep (including latency, inertia, and duration of sleep) could be taken into account. I suggest you rework on methodological errors to improve current form of manuscript. Study limitations should be adequately mentioned under relevant headings.

**[Response]**

**We thank the Editor for highlighting the importance of incorporating additional physiological and behavioral indicators such as skin temperature, ECG, HR, HRV, sleep parameters (latency, inertia, duration), and physical activity. We fully agree that these factors are essential for developing a more ecologically valid and comprehensive model of thermally induced fatigue in real-world settings. In the present study, our focus was to establish a baseline understanding of the psychophysiological burden under temperature step conditions in a controlled environment. Nevertheless, we acknowledge this limitation and have added corresponding statements in the new Limitation section of the revised manuscript to outline directions for future research (see lines 418–457).**

**Responses to Reviewer #1**

**Reviewer #1’s comments:**

Despite the insufficient sample size, the study design was sound and thoroughly examined. We recommend conducting this study over a longer period of time and with a larger sample size in order to examine the physiological and psychological impacts of several successive temperature increments.

**[Response]**

**We sincerely thank the reviewer for recognizing the strengths of our study design. We fully agree with the suggestion that conducting the study over a longer period and with a larger sample size would help clarify the physiological and psychological impacts of repeated temperature changes more robustly.**

**As this study was designed as a preliminary investigation conducted under controlled laboratory conditions, we focused on establishing a basic psychophysiological response model. In the revised manuscript, we have now included a Limitation section discussing this point and emphasizing the need for future studies with extended duration and broader sample populations (see lines 418–457).**

**Responses to Reviewer #2**

**Reviewer #2’s comments:**

The worldwide exposure of the humans exposed to daily temperature differences indoors and outdoors associated to health risks and fatigue is a thoughtful concept. The study needs phenomenal changes as the approach is quite limited/narrow. The study aimed to clarify the psychophysiological loads by repeated short-term temperature differences on n = 28 (14 males and 14 females) Japanese individuals. The study planned is good, but of very short duration as the data collected for each test is of only 183 minutes for 4 times. First the duration of the study should be more, may be at least for a week or so, secondly, the effects should be recorded not only in the test duration but also pre and post test, to prove the case.

The scale of physical fatigue is quite large (0-100), making the psychological measurements more subjective.

Besides the psychophysiological and physiological measurements like HR, HRV, skin temperature and ECG, parameters like activity and sleep (latency, inertia and sleep duration) should also be considered.

The statistics has been thoroughly performed, but is too complex to understand. The comparisons between the temperature- controlled and the treated groups would have made the study/statistics simple and easy to prove.

Overall the experimental design should be more elaborate in order to demonstrate good internal control and structure. However, the lack of contextual and participant-specific data, along with a few methodological omissions, has impacted the interpretability and external validity of the findings.

**[Response]**

**We thank the reviewer for the thorough review of our manuscript. We sincerely appreciate for recognizing our research concept as "thoughtful" and for providing numerous constructive comments.**

**We have addressed each of the concerns below and have made revisions throughout the manuscript based on your valuable feedback.**

1. **Study duration and measurement timing**

**We appreciate the reviewer’s thoughtful comments regarding the limited study duration and measurement design. This study was conducted under controlled laboratory conditions with the aim of exploring short-term psychophysiological responses to temperature steps. We fully agree that longer-term studies with pre- and post-assessments, as well as real-world observations, are essential for future work. We have added a new Limitation section to the manuscript addressing this issue (see lines 418–430).**

2. **Use of VAS for fatigue assessment
   We thank the reviewer’s comment on the use of a 0–100 visual analogue scale (VAS) for assessing physical fatigue. The VAS is a widely used and validated tool for capturing subjective fatigue, as shown by Lee et al. (1991). Its continuous format allows for detecting subtle changes that may be missed by categorical scales, making it suitable for psychophysiological studies like ours. To minimize variability, we gave participants clear instructions with anchor definitions (0 = no fatigue, 100 = complete exhaustion) and ensured consistency across sessions. We have also revised the Methods section to better explain our use of the VAS (see lines 112–118).**
3. **Consideration of additional parameters such as sleep and activity**

**We thank the reviewer for highlighting the importance of including additional physiological and behavioral factors such as activity and sleep (latency, inertia, and duration). We agree that incorporating these variables would enhance the ecological validity of future models. In this study, our focus was to establish a psychophysiological load model within a controlled setting. We have now noted the absence of these variables and the need for more comprehensive assessments in future studies in the Limitation section (see lines 431–437).**

4. **Statistical methodology
   We appreciate the reviewer’s feedback regarding the complexity of the statistical approach. To address our primary goal—capturing the temporal dynamics of psychophysiological load—we employed a Bayesian state-space model. This method is particularly suited for time-series data with repeated measures, where each observation is autocorrelated with prior states.**

**Unlike conventional methods that assume independence between time points, the state-space model distinguishes between the latent internal state (e.g., underlying fatigue) and random observation noise, allowing us to track how participants’ internal states evolve over time. This approach provides a more accurate assessment of intervention effects than simple group-level comparisons at discrete time points.**

**We also included a temperature-stable condition (26°C–26°C) as a control group for comparison.**
